# Poly[[(2,2′-bipyridine)­(μ_3_-2-sulfonatobenzoato)lead(II)] dihydrate]

**DOI:** 10.1107/S1600536810005763

**Published:** 2010-02-17

**Authors:** Li-Qing Yang, Xin-Hua Li

**Affiliations:** a College of Chemistry and Materials Engineering, Wenzhou University, Wenzhou 325035, People’s Republic of China

## Abstract

In the title compound, {[Pb(sbc)(bpy)]·2H_2_O}_*n*_ [bpy is 2,2′-bipyridine (C_10_H_8_N_2_) and sbc is the 2-sulfonatobenzoate dianion (C_7_H_4_O_5_S)], the Pb^II^ ion is bonded to four O atoms including carboxyl­ate and sulfonate from three sbc dianions, and two N atoms from a chelating 2,2′-bipyridine ligand. The sbc ligand acts as a μ_3_-bridging ligand by one O atom of the sulfonate group and the two O atoms of the carboxyl­ate. Of these two last O atoms, one builds up a dinuclear framework arranged around an inversion center whereas the second one links each dinuclear unit, forming a chain extending along the *b* axis. These polymeric chains are linked through O—H⋯O hydrogen bonds involving the water mol­ecules, forming a layer parallel to (10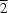).

## Related literature

For general background to lead coordination modes, see: Bridgewater & Parkin (2000 ▶); Cecconi *et al.* (2003 ▶); Taheri & Morsali (2006 ▶); Wang & Vittal (2003 ▶); Yin & Yu (2007 ▶); Foreman *et al.* (2000 ▶). For coordination based on sbc ligands, see: Xiao (2006 ▶); Xiao *et al.* (2005 ▶, 2008 ▶); Ying *et al.* (2003 ▶); Li *et al.* (2008 ▶); Shi *et al.* (2007 ▶). For information on sulfonate geometry, see: Onoda *et al.* (2001 ▶).
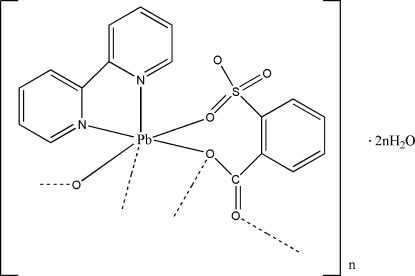


## Experimental

### 

#### Crystal data

[Pb(C_7_H_4_O_5_S)(C_10_H_8_N_2_)]·2H_2_O
*M* *_r_* = 599.57
Monoclinic, 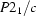

*a* = 15.3464 (11) Å
*b* = 6.9951 (5) Å
*c* = 17.2844 (12) Å
β = 96.629 (1)°
*V* = 1843.1 (2) Å^3^
*Z* = 4
Mo *K*α radiation
μ = 9.31 mm^−1^
*T* = 298 K
0.50 × 0.21 × 0.15 mm

#### Data collection

Bruker SMART CCD area-detector diffractometer
Absorption correction: multi-scan (*SADABS*; Bruker, 2002 ▶) *T*_min_ = 0.11, *T*_max_ = 0.26
9382 measured reflections
3318 independent reflections
2944 reflections with *I* > 2σ(*I*)
*R*_int_ = 0.029

#### Refinement

*R*[*F* ^2^ > 2σ(*F* ^2^)] = 0.023
*wR*(*F* ^2^) = 0.058
*S* = 1.04
3318 reflections
253 parameters
H-atom parameters constrained
Δρ_max_ = 0.91 e Å^−3^
Δρ_min_ = −0.83 e Å^−3^

### 

Data collection: *SMART* (Bruker, 2002 ▶); cell refinement: *SAINT* (Bruker, 2002 ▶); data reduction: *SAINT*; program(s) used to solve structure: *SHELXS97* (Sheldrick, 2008 ▶); program(s) used to refine structure: *SHELXL97* (Sheldrick, 2008 ▶); molecular graphics: *PLATON* (Spek, 2009 ▶) and *XP* in *SHELXTL* (Sheldrick, 2008 ▶); software used to prepare material for publication: *SHELXL97*.

## Supplementary Material

Crystal structure: contains datablocks I, global. DOI: 10.1107/S1600536810005763/dn2537sup1.cif

Structure factors: contains datablocks I. DOI: 10.1107/S1600536810005763/dn2537Isup2.hkl

Additional supplementary materials:  crystallographic information; 3D view; checkCIF report

## Figures and Tables

**Table 1:** Hydrogen-bond geometry (Å, °)

*D*—H⋯*A*	*D*—H	H⋯*A*	*D*⋯*A*	*D*—H⋯*A*
O6—H6*A*⋯O4^i^	0.85	1.97	2.808 (5)	168
O6—H6*B*⋯O7^ii^	0.85	1.90	2.752 (6)	178
O7—H7*A*⋯O4	0.85	1.95	2.791 (5)	169
O7—H7*B*⋯O6	0.85	1.89	2.722 (6)	167

Symmetry codes: (i) 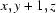; (ii) 
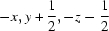
.

**Fig. 1.**
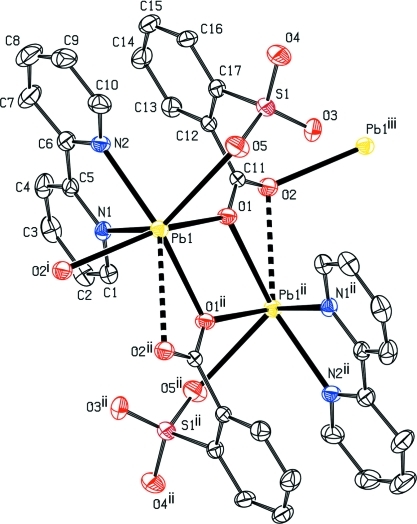
The coordination environment of lead (II) ion in (I) with the atom labeling scheme. Ellipsoids are drawn at the 30% probability level. Water molecules and H atoms have been omitted for clarity. [Symmetry codes: (i) x, y+1, z; (ii) -x+1, -y, -z; (iii) x, y-1, z]

**Fig. 2.**
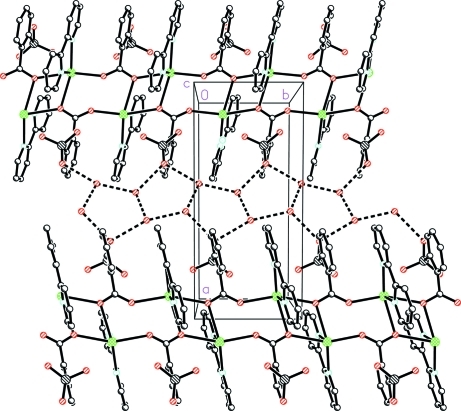
View showing the O-H···O bond network built up by the water molecules intercalated between the polymeric chains. H atoms not involved in hydrogen bondings have been omitted for clarity.

## supplementary crystallographic information

### Comment

Lead(II) is capable of exhibiting variable coordination mode forming a range of coordination polymers and polynuclear complexes geometry (Wang & Vittal, 2003; Cecconi *et al.*, 2003; Bridgewater *et al.*, 2000, Ying *et al.*, 2003; Taheri & Morsali, 2006; Yin & Yu, 2007). The absence of crystal field stabilisation energy effects also allows the Pb(II) cations to adopt a range of different coordination geometries not restricted to octahedral, tetrahedral or square planar (Foreman *et al.*, 2000). Sbc is an interesting ligand with both carboxylate and sulfonate acting as potential coordinating groups. Some metal-organic coordinations based on Sbc ligand have been reported (Li *et al.*, 2008, Xiao *et al.*, 2005, Xiao *et al.*, 2006, Xiao *et al.*, 2008, Shi *et al.*, 2007). Thus, we have selected the Pb-sbc system to extend our research and we present here the crystal structure of the title compound, [Pb(sbc)(bpy)]^.^2H_2_O (bpy is 2,2'-bipyridine and sbc is 2-sulfobenzenecarboxylate dianion), (I).

The Pb atom might be regarded as six or seven coordinates if the second carboxylate O atom is considered as weakly bonding to the metal as observed in the related compound (C_34_ H_20_ N_2_ O_8_ Pb2)n (Yin & Yu, 2007) (Fig. 1). The Pb1—O2(symmetry code: ), 3.045 \%A, is much longer than the 2.745 \%A reported in the related complex, but it is still shorter than the sum of the Van der Waals radii. The geometry around the metal might be described as highly distorted monocaped octahedron.

The sbc ligand acts as a µ_3_-bridging ligands by one O atom of the sulfone group, and the two O atoms of the carboxylate. Of these two last O atoms, one is building a dinuclear framework arranged around inversion center whereas the second one is linking each dinuclear unit to form a chain developping along the b axis.(Fig.2).

Interestingly, the water molecules are intercalated between the polymeric chains and link these chains through O-H^···^O hydrogen bonds to build up layers developping parallel to the (1 0 -2) plane (Table 1, Fig. 2).

The S-O distances within the sulfonate fall within the typical range observed for S-O bonds (Onoda *et al.*, 2001). The similarity of the three S—O bond distances suggests that strong conjugation on sulfonate is predominant in (I).

### Experimental

The title compound was synthesized by adding the DMF solution (10 ml) of 2,2'-bipyridine (0.03 g, 0.2 mmol) and 2,2'-dithiosalicylic acid (0.06 g, 0.2 mmol) dropwise to a stirred water solution (10 ml) of lead nitrate (0.07 g, 0.2 mmol) at 298 K temperature. Then the reaction mixture was filtered and the filtrate stood for about six weeks until the prism colorless crystals were obtained. The prism shaped crystals suitable for X-ray diffraction were collected by filtration, washed with water and ethanol and dried in air. The structure of (I) was determined by single crystal X-ray crystallography. Intensity data and unit-cell parameters for (I) were measured at 298 K on a Bruker Smart 1000 CCD diffractometer with graphite-monochromated Mo Kα radiation (λ=0.71073 Å) and a graphite monochromator using the ω-scan mode. All empirical absorption corrections were applied by using the SADABS program[Bruker, 2002]. The structure was solved by direct methods and refined on F^2^ by full-matrix leastsquares using the SHELXL-97 program package[Bruker, 2002].

### Refinement

The water H atoms were refined subject to the restraint O—H = 0.82 (5) Å. The other H atoms were positioned geometrically and allowed to ride on their parent atoms at distances of 0.93 Å with U_iso_= 1.2U_eq_(parent atom).

### Crystal data

**Table:**

[Pb(C_7_H_4_O_5_S)(C_10_H_8_N_2_)]·2H_2_O	*F*(000) = 1144
*M**_r_* = 599.57	*D*_x_ = 2.161 Mg m^−^^3^
Monoclinic, *P*2_1_/*c*	Mo *K*α radiation, λ = 0.71073 Å
Hall symbol: -P 2ybc	Cell parameters from 3318 reflections
*a* = 15.3464 (11) Å	θ = 2.4–25.2°
*b* = 6.9951 (5) Å	µ = 9.31 mm^−^^1^
*c* = 17.2844 (12) Å	*T* = 298 K
β = 96.629 (1)°	Prism, colorless
*V* = 1843.1 (2) Å^3^	0.50 × 0.21 × 0.15 mm
*Z* = 4	

### Data collection

**Table:**

Bruker SMART CCD area-detector diffractometer	3318 independent reflections
Radiation source: fine-focus sealed tube	2944 reflections with *I* > 2σ(*I*)
graphite	*R*_int_ = 0.029
600 frames, delta ω = 2 dgr scans	θ_max_ = 25.2°, θ_min_ = 2.4°
Absorption correction: multi-scan (*SADABS*; Bruker, 2002)	*h* = −18→13
*T*_min_ = 0.11, *T*_max_ = 0.26	*k* = −8→8
9382 measured reflections	*l* = −20→19

### Refinement

**Table:**

Refinement on *F*^2^	Primary atom site location: structure-invariant direct methods
Least-squares matrix: full	Secondary atom site location: difference Fourier map
*R*[*F*^2^ > 2σ(*F*^2^)] = 0.023	Hydrogen site location: inferred from neighbouring sites
*wR*(*F*^2^) = 0.058	H-atom parameters constrained
*S* = 1.03	*w* = 1/[σ^2^(*F*_o_^2^) + (0.0286*P*)^2^] where *P* = (*F*_o_^2^ + 2*F*_c_^2^)/3
3318 reflections	(Δ/σ)_max_ = 0.001
253 parameters	Δρ_max_ = 0.91 e Å^−^^3^
0 restraints	Δρ_min_ = −0.83 e Å^−^^3^

### Special details

**Table:**

Geometry. All esds (except the esd in the dihedral angle between two l.s. planes) are estimated using the full covariance matrix. The cell esds are taken into account individually in the estimation of esds in distances, angles and torsion angles; correlations between esds in cell parameters are only used when they are defined by crystal symmetry. An approximate (isotropic) treatment of cell esds is used for estimating esds involving l.s. planes.
Refinement. Refinement of *F*^2^ against ALL reflections. The weighted *R*-factor wR and goodness of fit *S* are based on *F*^2^, conventional *R*-factors *R* are based on *F*, with *F* set to zero for negative *F*^2^. The threshold expression of *F*^2^ > σ(*F*^2^) is used only for calculating *R*-factors(gt) etc. and is not relevant to the choice of reflections for refinement. *R*-factors based on *F*^2^ are statistically about twice as large as those based on *F*, and *R*- factors based on ALL data will be even larger.

### Fractional atomic coordinates and isotropic or equivalent isotropic displacement parameters (Å^2^)

**Table:**

	*x*	*y*	*z*	*U*_iso_*/*U*_eq_	
Pb1	0.402858 (9)	0.234749 (19)	−0.017420 (8)	0.03015 (7)	
S1	0.25615 (7)	−0.17058 (17)	−0.11614 (6)	0.0407 (3)	
O1	0.43147 (15)	−0.1252 (4)	0.00541 (15)	0.0333 (6)	
O2	0.42527 (16)	−0.4252 (4)	0.04577 (16)	0.0393 (7)	
O3	0.3148 (2)	−0.3281 (5)	−0.12513 (18)	0.0558 (8)	
O4	0.1728 (2)	−0.1882 (5)	−0.16536 (18)	0.0566 (8)	
O5	0.29504 (18)	0.0181 (4)	−0.12329 (15)	0.0503 (8)	
N1	0.4155 (2)	0.1825 (5)	0.12289 (17)	0.0307 (7)	
N2	0.2610 (2)	0.2622 (4)	0.0397 (2)	0.0351 (8)	
C1	0.4933 (2)	0.1312 (5)	0.1606 (2)	0.0352 (9)	
H1	0.5424	0.1327	0.1337	0.042*	
C2	0.5031 (3)	0.0765 (6)	0.2376 (2)	0.0443 (10)	
H2	0.5576	0.0398	0.2622	0.053*	
C3	0.4304 (3)	0.0773 (6)	0.2774 (2)	0.0499 (11)	
H3	0.4351	0.0400	0.3294	0.060*	
C4	0.3511 (3)	0.1333 (6)	0.2399 (2)	0.0476 (11)	
H4	0.3018	0.1355	0.2665	0.057*	
C5	0.3442 (3)	0.1870 (5)	0.1619 (2)	0.0340 (9)	
C6	0.2604 (3)	0.2435 (5)	0.1169 (3)	0.0382 (11)	
C7	0.1840 (4)	0.2756 (6)	0.1507 (4)	0.0584 (15)	
H7	0.1837	0.2637	0.2042	0.070*	
C8	0.1089 (3)	0.3251 (8)	0.1042 (4)	0.0723 (17)	
H8	0.0572	0.3459	0.1262	0.087*	
C9	0.1097 (3)	0.3439 (7)	0.0258 (4)	0.0638 (15)	
H9	0.0592	0.3776	−0.0063	0.077*	
C10	0.1878 (3)	0.3116 (6)	−0.0047 (3)	0.0500 (12)	
H10	0.1892	0.3248	−0.0581	0.060*	
C11	0.3901 (3)	−0.2673 (5)	0.0288 (2)	0.0274 (9)	
C12	0.2956 (3)	−0.2376 (4)	0.0411 (3)	0.0308 (9)	
C13	0.2730 (3)	−0.2575 (5)	0.1167 (3)	0.0389 (11)	
H13	0.3152	−0.2949	0.1568	0.047*	
C14	0.1877 (3)	−0.2215 (6)	0.1319 (3)	0.0491 (13)	
H14	0.1731	−0.2319	0.1825	0.059*	
C15	0.1244 (3)	−0.1702 (6)	0.0723 (3)	0.0477 (11)	
H15	0.0674	−0.1455	0.0829	0.057*	
C16	0.1451 (2)	−0.1554 (6)	−0.0030 (3)	0.0412 (10)	
H16	0.1019	−0.1232	−0.0431	0.049*	
C17	0.2304 (2)	−0.1884 (5)	−0.0192 (2)	0.0316 (8)	
O6	0.0809 (3)	0.4682 (6)	−0.1967 (3)	0.1033 (15)	
H6A	0.1149	0.5621	−0.1834	0.155*	
H6B	0.0420	0.5073	−0.2324	0.155*	
O7	0.0420 (2)	0.0904 (6)	−0.1850 (2)	0.0793 (11)	
H7A	0.0845	0.0127	−0.1844	0.119*	
H7B	0.0594	0.2030	−0.1938	0.119*	

### Atomic displacement parameters (Å^2^)

**Table:**

	*U*^11^	*U*^22^	*U*^33^	*U*^12^	*U*^13^	*U*^23^
Pb1	0.02874 (11)	0.03411 (11)	0.02743 (11)	−0.00222 (5)	0.00244 (7)	0.00072 (6)
S1	0.0343 (5)	0.0518 (7)	0.0355 (6)	−0.0060 (5)	0.0012 (5)	−0.0130 (5)
O1	0.0276 (13)	0.0314 (15)	0.0411 (15)	−0.0041 (11)	0.0041 (12)	0.0006 (12)
O2	0.0319 (14)	0.0303 (16)	0.0554 (18)	0.0020 (11)	0.0035 (13)	−0.0029 (13)
O3	0.0476 (18)	0.063 (2)	0.058 (2)	0.0065 (16)	0.0098 (16)	−0.0280 (18)
O4	0.0419 (18)	0.078 (2)	0.0463 (19)	−0.0079 (16)	−0.0092 (15)	−0.0141 (17)
O5	0.0535 (17)	0.060 (2)	0.0376 (17)	−0.0182 (15)	0.0049 (14)	0.0002 (14)
N1	0.0368 (18)	0.0277 (17)	0.0291 (17)	−0.0016 (14)	0.0104 (14)	−0.0001 (14)
N2	0.0274 (18)	0.030 (2)	0.048 (2)	−0.0033 (12)	0.0056 (17)	−0.0017 (13)
C1	0.041 (2)	0.033 (2)	0.032 (2)	−0.0011 (17)	0.0041 (18)	−0.0015 (17)
C2	0.058 (3)	0.033 (2)	0.039 (3)	0.0010 (19)	−0.008 (2)	0.0034 (18)
C3	0.078 (3)	0.040 (3)	0.031 (2)	−0.006 (2)	0.008 (2)	0.0026 (18)
C4	0.066 (3)	0.043 (3)	0.039 (2)	−0.004 (2)	0.026 (2)	−0.002 (2)
C5	0.041 (2)	0.0252 (19)	0.038 (2)	−0.0063 (17)	0.0139 (19)	−0.0042 (17)
C6	0.040 (2)	0.023 (2)	0.054 (3)	−0.0020 (15)	0.014 (2)	−0.0020 (16)
C7	0.050 (3)	0.050 (3)	0.082 (4)	0.006 (2)	0.036 (3)	0.001 (2)
C8	0.046 (3)	0.054 (3)	0.123 (6)	0.009 (2)	0.036 (4)	−0.001 (4)
C9	0.035 (3)	0.039 (3)	0.116 (5)	0.004 (2)	0.004 (3)	−0.006 (3)
C10	0.036 (2)	0.039 (2)	0.073 (3)	0.001 (2)	−0.004 (2)	−0.004 (2)
C11	0.025 (2)	0.028 (2)	0.029 (2)	−0.0016 (14)	0.0022 (17)	−0.0058 (15)
C12	0.030 (2)	0.020 (2)	0.044 (3)	−0.0058 (13)	0.0117 (19)	−0.0062 (15)
C13	0.043 (3)	0.033 (3)	0.041 (3)	−0.0034 (16)	0.007 (2)	0.0032 (16)
C14	0.054 (3)	0.045 (3)	0.054 (3)	−0.008 (2)	0.031 (3)	−0.001 (2)
C15	0.034 (2)	0.043 (3)	0.069 (3)	−0.001 (2)	0.021 (2)	−0.004 (2)
C16	0.025 (2)	0.038 (2)	0.061 (3)	−0.0005 (17)	0.007 (2)	−0.004 (2)
C17	0.032 (2)	0.0246 (19)	0.039 (2)	0.0004 (16)	0.0069 (17)	−0.0022 (17)
O6	0.112 (3)	0.067 (3)	0.119 (4)	−0.020 (2)	−0.037 (3)	0.005 (2)
O7	0.056 (2)	0.075 (3)	0.105 (3)	0.0065 (18)	0.002 (2)	0.012 (2)

### Geometric parameters (Å, °)

**Table:**

Pb1—N1	2.438 (3)	C5—C6	1.477 (6)
Pb1—N2	2.499 (4)	C6—C7	1.387 (7)
Pb1—O1	2.579 (3)	C7—C8	1.371 (8)
Pb1—O2^i^	2.623 (3)	C7—H7	0.9300
Pb1—O1^ii^	2.640 (2)	C8—C9	1.362 (7)
S1—O3	1.442 (3)	C8—H8	0.9300
S1—O4	1.457 (3)	C9—C10	1.382 (6)
S1—O5	1.460 (3)	C9—H9	0.9300
S1—C17	1.770 (4)	C10—H10	0.9300
O1—C11	1.270 (4)	C11—C12	1.503 (5)
O1—Pb1^ii^	2.640 (2)	C12—C13	1.397 (7)
O2—C11	1.250 (4)	C12—C17	1.402 (6)
O2—Pb1^iii^	2.623 (3)	C13—C14	1.389 (7)
N1—C1	1.341 (5)	C13—H13	0.9300
N1—C5	1.350 (5)	C14—C15	1.380 (7)
N2—C10	1.331 (6)	C14—H14	0.9300
N2—C6	1.343 (6)	C15—C16	1.379 (6)
C1—C2	1.377 (5)	C15—H15	0.9300
C1—H1	0.9300	C16—C17	1.388 (5)
C2—C3	1.376 (5)	C16—H16	0.9300
C2—H2	0.9300	O6—H6A	0.8533
C3—C4	1.369 (6)	O6—H6B	0.8532
C3—H3	0.9300	O7—H7A	0.8484
C4—C5	1.392 (5)	O7—H7B	0.8510
C4—H4	0.9300		
			
N1—Pb1—N2	65.91 (11)	C4—C5—C6	123.1 (4)
N1—Pb1—O1	73.07 (9)	N2—C6—C7	120.3 (5)
N2—Pb1—O1	98.94 (8)	N2—C6—C5	116.4 (4)
N1—Pb1—O2^i^	74.35 (9)	C7—C6—C5	123.3 (5)
N2—Pb1—O2^i^	81.04 (9)	C8—C7—C6	119.3 (6)
O1—Pb1—O2^i^	144.08 (8)	C8—C7—H7	120.4
N1—Pb1—O1^ii^	85.01 (9)	C6—C7—H7	120.4
N2—Pb1—O1^ii^	150.00 (10)	C9—C8—C7	120.2 (5)
O1—Pb1—O1^ii^	63.85 (9)	C9—C8—H8	119.9
O2^i^—Pb1—O1^ii^	98.72 (7)	C7—C8—H8	119.9
O3—S1—O4	112.87 (19)	C8—C9—C10	118.2 (5)
O3—S1—O5	114.6 (2)	C8—C9—H9	120.9
O4—S1—O5	111.53 (19)	C10—C9—H9	120.9
O3—S1—C17	104.97 (19)	N2—C10—C9	122.2 (5)
O4—S1—C17	105.67 (19)	N2—C10—H10	118.9
O5—S1—C17	106.38 (17)	C9—C10—H10	118.9
C11—O1—Pb1	136.9 (2)	O2—C11—O1	123.2 (4)
C11—O1—Pb1^ii^	105.1 (2)	O2—C11—C12	119.1 (3)
Pb1—O1—Pb1^ii^	116.15 (9)	O1—C11—C12	117.6 (3)
C11—O2—Pb1^iii^	132.2 (2)	C13—C12—C17	119.1 (4)
C1—N1—C5	119.4 (3)	C13—C12—C11	117.8 (4)
C1—N1—Pb1	119.2 (2)	C17—C12—C11	123.2 (4)
C5—N1—Pb1	121.1 (3)	C14—C13—C12	120.1 (5)
C10—N2—C6	119.8 (4)	C14—C13—H13	119.9
C10—N2—Pb1	120.6 (3)	C12—C13—H13	119.9
C6—N2—Pb1	119.4 (3)	C15—C14—C13	120.2 (4)
N1—C1—C2	122.4 (4)	C15—C14—H14	119.9
N1—C1—H1	118.8	C13—C14—H14	119.9
C2—C1—H1	118.8	C16—C15—C14	120.3 (4)
C3—C2—C1	118.5 (4)	C16—C15—H15	119.8
C3—C2—H2	120.7	C14—C15—H15	119.8
C1—C2—H2	120.7	C15—C16—C17	120.2 (4)
C4—C3—C2	119.5 (4)	C15—C16—H16	119.9
C4—C3—H3	120.3	C17—C16—H16	119.9
C2—C3—H3	120.3	C16—C17—C12	120.0 (4)
C3—C4—C5	120.0 (4)	C16—C17—S1	119.8 (3)
C3—C4—H4	120.0	C12—C17—S1	120.1 (3)
C5—C4—H4	120.0	H6A—O6—H6B	107.6
N1—C5—C4	120.1 (4)	H7A—O7—H7B	109.7
N1—C5—C6	116.7 (4)		

Symmetry codes: (i) *x*, *y*+1, *z*; (ii) −*x*+1, −*y*, −*z*; (iii) *x*, *y*−1, *z*.

### Hydrogen-bond geometry (Å, °)

**Table:**

*D*—H···*A*	*D*—H	H···*A*	*D*···*A*	*D*—H···*A*
O6—H6A···O4^i^	0.85	1.97	2.808 (5)	168
O6—H6B···O7^iv^	0.85	1.90	2.752 (6)	178
O7—H7A···O4	0.85	1.95	2.791 (5)	169
O7—H7B···O6	0.85	1.89	2.722 (6)	167

Symmetry codes: (i) *x*, *y*+1, *z*; (iv) −*x*, *y*+1/2, −*z*−1/2.
